# Preaugmentation Soft Tissue Expansion: A Report of Four Pilot Cases

**DOI:** 10.1155/2018/3162617

**Published:** 2018-04-19

**Authors:** Farah Asa'ad, Gionata Bellucci, Luca Ferrantino, Davide Trisciuoglio, Silvio Taschieri, Massimo Del Fabbro

**Affiliations:** ^1^Department of Biomedical, Surgical and Dental Sciences, Foundation IRCCS Ca' Granda Polyclinic, University of Milan, Milan, Italy; ^2^Department of Biomedical, Surgical and Dental Sciences, IRCCS Galeazzi Orthopaedic Institute, University of Milan, Milan, Italy

## Abstract

This pilot study aimed at investigating the safety and feasibility of pre-augmentation soft tissue expansion (STE). Tissue expanders of different sizes (from 240 to 1300 mm^3^) were implanted subperiosteally in four patients requiring vertical and/or horizontal bone augmentation, and left in situ for 20–60 days, according to the expander size. Guided bone regeneration was carried out after STE completion. Horizontal and vertical bone gains were analyzed through CBCT. Optical scanning and superimposition of cast models were used for volumetric analysis. The mean soft tissue volume increase was 483.8 ± 251.7 mm^3^. Horizontal bone gain averaged 3 mm in two successfully expanded sites while one case had a vertical bone gain of 8 mm. Despite promising outcomes in bone and soft tissue gain, the present technique needs improvement before being applied routinely in everyday dental practice.

## 1. Introduction

In modern dental practice, placement of endosseous implants is constantly increasing, as many patients are seeking replacement of lost teeth with this modality of treatment. Since the overall success of dental implant therapy depends on the presence of adequate bone volume at implant sites [1], sufficient vertical and horizontal amounts of alveolar ridge prior to dental implant placement are essential.

Bone augmentation can be performed using different techniques: bone blocks and/or guided bone regeneration (GBR) are applied for horizontal bone augmentation [2]. Vertical bone augmentation employs more challenging and technique-sensitive methods: vertical GBR, onlay grafting, inlay grafting, and distraction osteogenesis [3, 4], and is frequently associated with high complication rates such as soft tissue dehiscence and subsequent exposure of bone grafts into the oral cavity [5].

Consequently, soft tissue expanders have been introduced in implant therapy, as pre-augmentation devices, to avoid the complications associated with bone-grafting procedures [6–9]. The currently used soft tissue expanders made of hydrogel, which is the same material used to fabricate contact lenses, are designed and manufactured since 1999 under the name of Osmed® (Ilmenau, Germany), which is the first commercially available self-inflatable osmotic expander and has been FDA-approved since 2001.

Up to date, there is scarce clinical evidence describing soft tissue expansion (STE) prior to bone augmentation procedures: only two case series [6, 8] and one randomized controlled clinical trial [7] are available in literature. These studies have evaluated the outcomes of bone regeneration, but neither has provided clear technical guidelines on the intraoral clinical utilization of these devices nor volumetric analysis of soft tissues. Only post-expansion changes in the profile of the attached gingiva was evaluated in one randomized controlled clinical trial [7]. The authors did not measure the total volume change of soft tissues, as they only aimed to determine the overall stability of the expanded soft tissues by evaluating their profile changes over time.

Based on these observations, we present a report of four pilot cases on preaugmentation soft tissue expansion, utilizing Osmed® expanders (Osmed GmbH, Ilmenau, Germany), to gain insight into the safety and effectiveness of this approach. We also performed volumetric analysis by optic scanning to evaluate the changes in soft tissue volume post-expansion.

## 2. Materials and Methods

This clinical study was conducted in the period between May 2016 and September 2017.

### 2.1. Study Participants and Inclusion Criteria

From the pool of patients attending the Dental Clinic of the Ospedale Maggiore Policlinico, University of Milan, Milan, Italy, four participants requiring alveolar bone augmentation and dental implant placement were included in this clinical investigation. All patients were enrolled into the study after explaining its objectives and obtaining their verbal and written informed consent. All patients were treated according to the principles enunciated in the Helsinki Declaration of 1980 for biomedical research involving human subjects.

Study participants fit the following inclusion criteria:

1. Patients in need for bone augmentation procedures in vertical and/or horizontal dimensions, either in the maxilla or mandible, prior to dental implant placement.
2. The edentulous area of interest had insufficient amount of soft tissues.
3. In partially edentulous areas, neighboring teeth should have had no clinical signs of caries, periapical infections, or periodontal inflammation. If active periodontal disease was present, the periodontal condition had to be stabilized first.
4. Patients without any systemic diseases (ASA-1 or ASA-2 according to the classification of the American Society of Anaesthesiologists).
5. Non-smokers or ex-smokers who have quit smoking since at least one-year prior to enrollment in the study.

The exclusion criteria were the following:

1. Self-declaration of pregnancy
2. Patients on medications that would adversely affect the outcomes of implant therapy and bone regeneration procedures (e.g., bisphosphonates and antiresorptive drugs)

## 3. Case Presentation

### 3.1. Case 1

A 44-year-old female patient of Caucasian origin visited the dental clinic seeking replacement of missing teeth in the lower right mandible. Clinical examination revealed missing right mandibular 1st, 2nd, and 3rd molars. Severe bone resorption was evident, accompanied by inadequate amounts of soft tissue. Radiographic evaluation on cone beam computed tomography (CBCT) scans revealed severe vertical bone resorption. Based on these findings, pre-augmentation soft tissue expansion (STE) was scheduled, followed by vertical bone augmentation and placement of dental implants.

### 3.2. Case 2

A 53-year-old male patient of Caucasian origin visited the dental clinic to substitute a removable acrylic partial denture that he has been wearing for over five years with a fixed prosthesis in the lower jaw. After the patient was asked to remove the denture, clinical examination revealed that all lateral and central incisors were missing. Signs of bone resorption and inadequate amounts of soft tissue were clearly visible. CBCT scan revealed severe horizontal bone resorption. In order to install implant-supported fixed prosthesis, pre-augmentation STE was planned as the first step, followed by horizontal bone augmentation and dental implant placement.

### 3.3. Case 3

A 58-year-old female patient of Caucasian origin visited the dental clinic to replace a missing lower mandibular first molar. Dental history revealed that the missing tooth was already restored with a dental implant one year ago, but the implant has failed and was removed few months ago. Signs of bone resorption and inadequate soft tissue amount were obvious upon clinical examination. CBCT scan revealed severe horizontal bone resorption. Preaugmentation STE was scheduled, followed by horizontal bone augmentation and dental implant placement.

### 3.4. Case 4

A 60-year-old female patient of Caucasian origin visited the dental clinic to replace missing teeth in the upper right and left posterior maxillae. Clinical examination of the upper right posterior maxilla revealed missing 2nd premolar and 1st molar, while all the posterior maxillary teeth were completely missing in the left side (i.e., 1st and 2nd premolars and 1st, 2nd, and 3rd molars). Soft tissue amount was inadequate in both areas. CBCT scan showed a moderate horizontal bone loss in both sides. Therefore, STE in the right and left maxillary sides followed by horizontal bone augmentation and dental implant placement were planned.

All the photos present in this report represent the left maxillary side of Case 4.

### 3.5. Implantation of Soft Tissue Expanders

**Figure 1:**
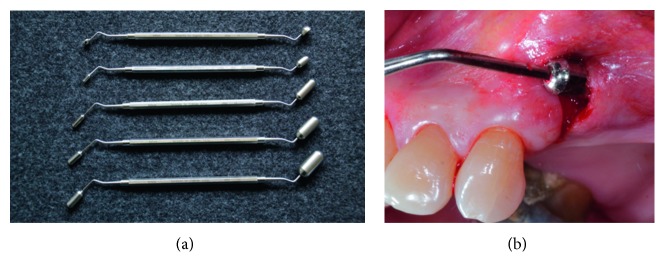
(a) Specific surgical templates used to choose the appropriate soft tissue expander. Each template has two ends that reflect the initial and final expander volumes. Courtesy of Osmed GmbH (Ilmenau, Germany). (b) Subperiosteal pouch prepared under local anesthesia and controlled with the surgical template.

Based on the extension and location of the edentulous area, intraoral cupola expanders (final volume: 0.35 ml) or cylinder expanders (final volumes: 0.24 ml, 0.7 ml, and 1.3 ml) were applied (Osmed expanders, Osmed GmbH, Ilmenau, Germany). Expanders were left in situ for 20, 40, or 60 days, depending on the final volume of the utilized expander. The appropriate expander was selected using a specific surgical template corresponding to the initial and final volumes of the expander (Figure 1(a)).

**Figure 2:**
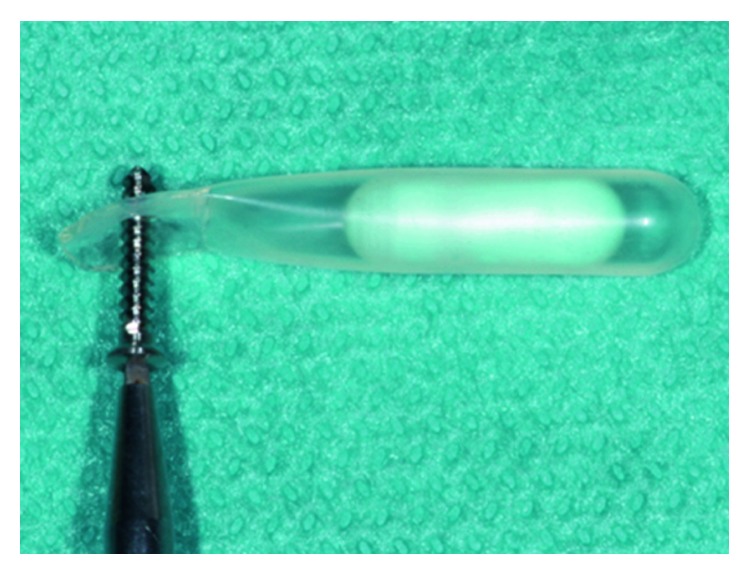
Insertion of bone fixation screw at the flat end to prevent potential expander migration.

Expanders were inserted using the same surgical technique previously described in literature [8]. Briefly, expanders were inserted in a subperiosteal pouch prepared under local anesthesia and controlled with the specific surgical template (Figure 1(b)) to ensure the device is easily fit without tension into the prepared pouch. The expander was handled carefully by holding its flat end with tweezer. To prevent any dislocation or potential migration, expanders were fixed with a bone fixation screw (Figure 2), at the flat end, which does not have an expansion capability. In all cases, the surgical site was closed utilizing a mattress suture. No antibiotics were prescribed, and sutures were removed 10 days after expander insertion. Any complications, such as expander expulsion, and soft tissue changes in terms of color, inflammation, and bleeding were documented throughout the expansion period.

### 3.6. Expander Removal and Bone Augmentation

When the expansion phase was successfully completed, expander removal and bone augmentation were scheduled at the same appointment. Depending on the dimension of alveolar bone resorption, vertical and/or horizontal bone augmentation was performed.

**Figure 3:**
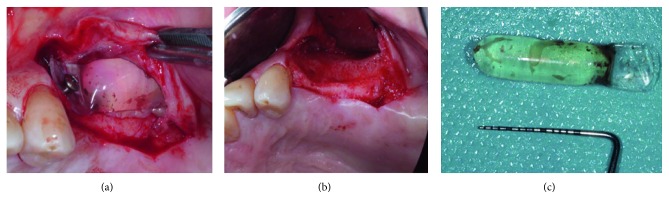
(a) Full-thickness flap was elevated to expose the expander and its fixation screw for removal. (b) Expander was removed, and signs of bone resorption due to expansion pressure are evident. (c) Cylinder expander of 0.7 ml final volume is removed. Fluid inside the expander is evident.

Under local anesthesia, a midcrestal incision was done and a full mucoperiosteal flap was released, and the expander and its fixing screw were removed. Bone surface was carefully examined for any signs of potential resorption due to pressure from the expander (Figures 3(a)–3(c)).

**Figure 4:**
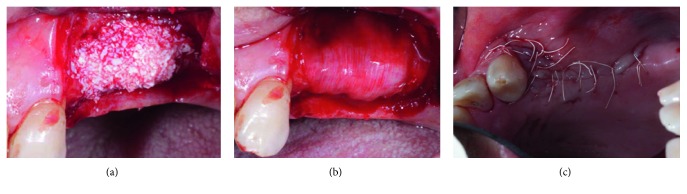
(a) Horizontal augmentation was done in this case, utilizing xenograft with autogenous bone. (b) Bone graft was covered with collagen absorbable membrane. (c) Primary, passive wound closure was achieved without deep periosteal releasing incisions.

In all cases, bone augmentation was performed using particulate autogenous bone harvested with bone scraper from the surgical site, mixed with xenograft (Bio-Oss®, Geistlich Pharma, Wolhusen, Switzerland). In case of vertical bone augmentation, the graft was covered with titanium reinforced PTFE high-density membrane (Cytoplast® Ti-250, Osteogenics Biomedical Inc., Lubbock, TX, USA), while collagen membrane was used (Bio-Gide®, Geistlich Pharma, Wolhusen, Switzerland), in the case of horizontal bone augmentation (Figures 4(a) and 4(b)). Tension-free primary closure was achieved in all cases without utilizing deep periosteal and/or vertical releasing incisions (Figure 4(c)).

For all patients, administration of antibiotics started one hour before the augmentation surgery (amoxicillin/calvulanic acid, 2 g) and continued for 7 days every 12 hours. Chlorhexidine mouthwash (0.2%) was recommended for daily use (3 times/day for 14 days). Ketoprofen (50 mg) was prescribed as an analgesic. Patients were followed up weekly, and sutures were removed two weeks after surgery. Any complications such as soft tissue dehiscence, membrane exposure, and bone graft expulsion were documented throughout the bone healing period.

### 3.7. Dental Implant Placement

**Figure 5:**
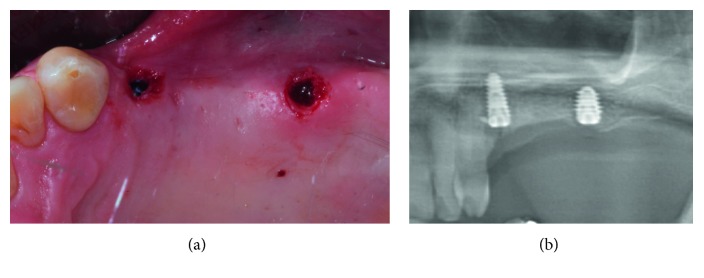
(a) Dental implant placement at the area that was expanded and augmented. (b) Section from panoramic radiograph representing the target site after dental implant placement.

In patients that completed the study, dental implants (MegaGen Implant Co., Ltd., Gyeongbuk, South Korea) were placed 6 months following bone augmentation (Figure 5(a)). All implants were submerged, and sutures were removed 7–14 days later.

### 3.8. Radiographs

Cone beam computed tomography (CBCT) scans were taken for all patients, before placement of soft tissue expanders and 4–6 months following bone augmentation procedures.

Soon after dental implant placement, intraoral radiographs with standardized, appropriate parameter settings, using the parallel technique and the proper film holders to ensure reproducibility, were taken for Case 1 and Case 3, while a panoramic radiograph was taken only for Case 4 (Figure 5(b)) as the patient in this case underwent dental implant placement in the left and right posterior maxillae at the same time.

Vertical and horizontal bone gains were calculated on CBCT scans, as previously described [6]. Briefly, subtraction of bone height or width “before augmentation” from bone height or width “before placement of dental implants” was performed at landmark sites using the CBCT software.

### 3.9. Volumetric Analysis by Optic Scanning

Volumetric analysis was performed using previously described methods [10, 11] with some modifications. First, alginate impressions were taken for each patient, one immediately before expander insertion and one when the expansion phase was successfully completed, that is, on the appointment of the expander removal and simultaneous bone augmentation. Then, two master casts, made of dental stone, were fabricated for each patient, based on the pre-expansion and post-expansion alginate impressions.

**Figure 6:**
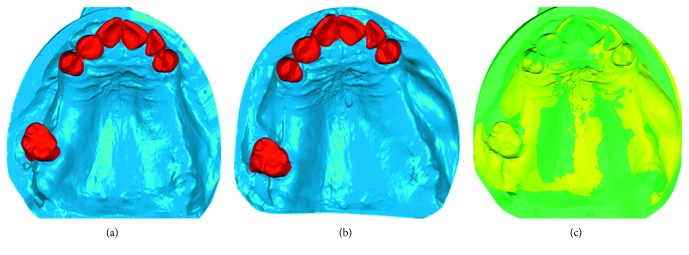
Volumetric analysis in the upper left maxilla. (a) Optically scanned preexpansion model. (b) Optically scanned postexpansion model. (c) Superimposed pre- and postexpansion models. Software used: Geomagic Studio 2013, Raindrop Geomagic, NC, USA.

**Figure 7:**
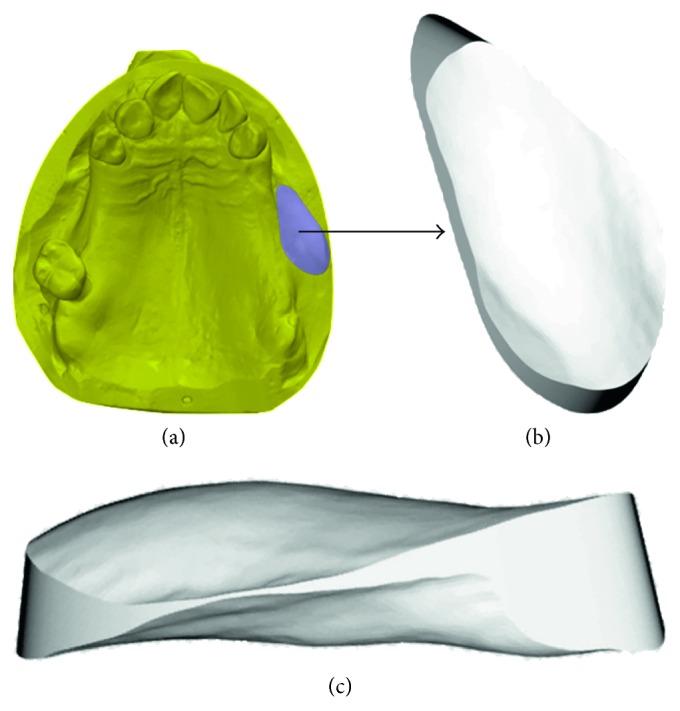
Calculation of volumetric changes. (a) Expanded tissues are highlighted with CAD software, and volume change is calculated. (b) The expanded area in STL format (coronal view), which can be superimposed on preexpansion STL file for further confirmation of calculation. (c) The expanded area in STL format (lateral view). Software used: Catia V5, Rand Worldwide Inc., Maryland, USA.

Next, volumetric changes of the soft tissues were assessed by using an optic scanner and two computer-aided design (CAD) software applications as follows: all the master casts were optically scanned with a 3D camera (Cerec 3D, Sirona Dental Systems GmbH, Bensheim, Germany) and digitalized creating STL files (Standard Tessellation Language). The STL files of pre- and postexpansion models were imported into CAD software (Geomagic Studio® 2013, Raindrop Geomagic, NC, USA) (Figures 6(a) and 6(b)). After being imported, files of pre- and postexpansion models for each patient were accurately superimposed, by using the buccal surface of adjacent teeth as a reference point (Figure 6(c)), applying the best-fit algorithm. After the superimposition was completed, by merging the pre- and postexpansion files into a one unique file, volume changes in the expansion area were calculated using another CAD software (Catia V5, Rand Worldwide Inc., Maryland, USA). The expanded tissues were highlighted with this CAD software, allowing for volume change calculation (Figure 7). After completing the calculations, the expanded area was then extracted into STL format, allowing for superimposition of this area over the original pre-expansion STL file for further confirmation (Figures 7 and 7). Volume analysis was done by the same calibrated examiner (FA).

## 4. Results

Four patients (3 females, 1 male, mean age = 53.6 ± 7.1 years, age range = 44–60 years) were included in this clinical pilot investigation. Expanders were placed at five surgical sites (in Case 4, two different expanders were placed in the same patient). One patient dropped out after soft tissue expansion has failed (Case 2); therefore, only three patients completed the scheduled treatment.

During the expansion period, healing was uneventful in 2 patients (“Case 1 and Case 4” at 3 surgical sites) and the soft tissues undergoing expansion did not show any signs of inflammation or bleeding during or after expansion was completed, while the expansion procedure failed in two patients (“Case 2 and Case 3” at 2 surgical sites) due to perforation of the expanders through the mucosa.

**Figure 8:**
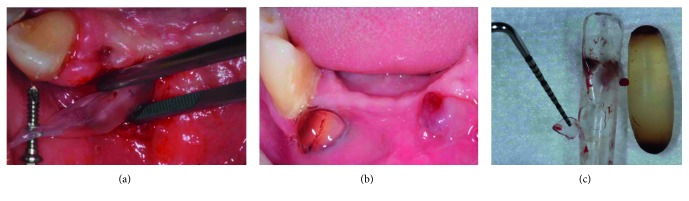
(a) The body of the expander was handled with dental tweezer during insertion, creating minor cracks. (b) Late failure of soft tissue expansion as seen by tissue perforation and expulsion of the expander. (c) Perforation of the silicon shell, due to propagation of the crack during expansion.

In one of these two sites, the expander was expelled due to crack formation of the silicon shell as a result of handling the body of the expander with the dental tweezer (Figures 8(a)–8(c), “Case 2”). It must be noted that the patient was wearing a removable partial denture during the expansion period despite being advised not to do so. Therefore, taking into consideration the patient's needs, the base of the denture was relieved to accommodate soft tissue expansion in the area. Nevertheless, it seems that wearing a denture, even if relieved, might have contributed to crack propagation in the silicon shell, eventually creating a perforation within the shell and subsequently causing expulsion of the expander at a very late stage of STE.

In the other failed site (Case 3), a cupola expander was inserted in a very tight mucosal pouch due to the anatomical location of the expansion site, which was the first molar. In this case, insertion of the expander in the classical horizontal direction was not possible due to the necessity to fix the expander at the flat end close to the mental nerve, so the prepared pouch was a bit tight to avoid any nerve injury. The expander was expelled within the first week of insertion.

Neither sites were retreated with expanders; one patient dropped out of the study (Case 2), and the other patient underwent bone augmentation two months after failed expansion.

In three patients, one site underwent vertical augmentation (Case 1) and three sites were regenerated horizontally (Case 3 and Case 4); two of the horizontally regenerated sites were preceded by successful STE (Case 4). Tension-free primary wound closure was easily achieved in the cases that were successfully treated with STE, without the need for periosteal deep incisions and/or vertical releasing incisions. It must be noted that deep periosteal releasing incisions were needed to advance the flap over the bone grafting material in the case of failed expansion (Case 3).

Following bone augmentation procedures, wound healing was uneventful, without any reported soft tissue dehiscence, graft expulsion, and/or membrane exposure.

Six months post-augmentation, CBCT analysis revealed that the vertical bone gain was 8 mm (Case 1), while horizontal bone gain for the two successfully expanded sites was 3 mm (Case 4). For the early failed soft tissue expansion case, horizontal bone gain was 2 mm (Case 3) (Table 1).

All three patients received dental implants in the augmented areas (one patient received one dental implant (Case 3), one patient received two dental implants (Case 1), and one patient received four dental implants (Case 4)). Diameter of placed implants ranged between 3.5 and 4 mm, while length range was 7–10 mm. All seven implants were successfully osseointegrated and scheduled for prosthetic rehabilitation. In those patients, no further soft tissue management was needed, even in terms of soft tissue augmentation.

### 4.1. Volumetric Analysis Results

Volumetric analysis was done for the three successfully expanded sites (Case 1 and Case 4). Regarding the failed cases, volumetric analysis was only done for the case in which late expansion fail occurred (Case 2), taking into consideration that the postexpansion alginate impression for this case was taken two weeks after failed expander removal.

Results of volumetric analysis are shown in Table 1. For the three successful expansion sites, the soft tissue volume increase was 259.4 mm^2^ for the 0.24 ml cylinder expander, 436.1 mm^2^ for the 0.7 ml cylinder expander, and 755.9 mm^2^ for the 1.3 ml cylinder expander (the mean volume increase of the three successful sites was 483.8 ± 251.7 mm^3^).

## 5. Discussion

In current literature, there are limited available clinical data that describe pre-augmentation soft tissue expansion: two case series [6, 8] and one randomized controlled clinical trial [7].

Kaner and Friedmann [6] were the first to describe the use of osmotic tissue expanders prior to vertical ridge augmentation, reporting a mean vertical bone gain at the time of dental implant placement of 7.5 ± 2.4 mm in twelve patients. In the present report, vertical bone augmentation was performed at one site only, showing a similar high vertical bone gain (8 mm). These findings might suggest that vertical bone augmentation preceded by STE could result in predictable vertical bone gain. In fact, a recent systematic review reported that mean vertical bone gain was 4.8 mm with classical bone augmentation procedures [5], which could highlight the importance of pre-augmentation STE.

In the present report, mean horizontal bone gain for successfully expanded sites was 3 mm, which is comparable to other findings in literature regarding bone gain following horizontal bone augmentation without preceding STE [12].

Surplus amount of soft tissues by STE allows for a passive primary closure of the flap minimizing postsurgical complications that would compromise bone fill, such as membrane and/or bone graft exposure. Interestingly, neither of these complications occurred in the current report of cases, and a very low incidence of graft exposure was reported by Kaner and Friedmann [6] (4%). When compared to other studies in literature, higher incidence of bone graft exposure was reported with vertical bone augmentation without preceding soft tissue expansion: 23% [13], 27.3% [14], 25% [15], 33.3% and 50% [16].

Despite the similar findings between the present report and the previously published case series [6], it must be noted that we did not exclusively investigate vertical bone augmentation. Furthermore, the method of expander insertion differed between both studies; we placed the expanders subperiosteally as we hypothesized it might be easier and less demanding surgically, while the submucosal approach has been advocated by Kaner and Friedmann [6] in an attempt to reduce the risk of bone resorption due to pressure exerted on bone surface by the expander. Nonetheless, signs of bone resorption after expander removal were evident on the bone surface at one site in the present report and at two sites in a different clinical study in which subperiosteal implantation of expanders was also employed [8].

In a randomized controlled clinical trial, no signs of bone resorption were reported with the subperiosteal approach which could be due to the much shorter duration of the expansion phase; expansion period of two weeks was chosen by the authors without following the manufacturer's guidelines, in order to avoid the formation of connective tissue capsule around the expander, which might replace the periosteum [7].

Complications related to osmotic tissue expanders reported in the literature have been attributed to different causes: infection, wearing a removable denture, expanding scarred tissues, and perforations either due to utilization of an excessively large expander or due to expander placement too close to the incision line [6–8]. In the present report, one expander failed because it was placed in a tight pouch due to anatomic considerations, and the other expander perforated the tissues at a very late stage into the expansion. Expander perforation into soft tissues at a very advanced stage of expansion has not been previously reported in literature. Therefore, we have looked carefully into the causes that might have contributed to this adverse event at a very late stage of expansion. Clinical photos taken during the surgical procedure revealed that the expander body, and not its flat end, was handled by a sharp instrument (dental tweezer). This might have led to the formation of a minor crack on the shell that propagated during the expansion phase, until the hydrogel body perforated through the crack all the way into the soft tissues.

Up to date, only one clinical study evaluated soft tissue changes during STE. In their randomized controlled clinical trial, Abrahamsson et al. [7] measured soft tissue stability of the attached gingiva at baseline and 6 months after augmentation in control and test groups and additionally at post-expansion in the test group, by using an objective 3D metering device. The mean soft tissue profile gain at the attached gingiva level was 2.9 ± 1.1 mm when compared to baseline, while it decreased to 2.3 ± 2.1 mm at the time of implant placement, when compared with baseline. The control group showed a soft profile change of 1.5 ± 1.4 mm at the time of fixture installation. Even if the test group showed increased gingival dimensions after surgeries, the differences were not statistically significant. The authors did not measure the total volume change in soft tissues, as they only wanted to determine overall stability of created soft tissues by evaluating soft tissue profile changes over time. Although soft tissue profile became less prominent after healing of bone graft when compared to pre-augmentation soft tissue profile, this result was statistically insignificant.

In attempt to evaluate the total volume change, we have done volumetric analysis using previously described methods [10, 11] with some modifications. For the three successful expansion sites, soft tissue volume increase corresponded only to the 0.24 ml (240 mm^3^) cylinder expander (volume increase = 259.4 mm^3^), while this increase was almost half of the final expander volume for the 0.7 ml (700 mm^3^) and 1.3 ml (1300 mm^3^) cylinder expanders (volume increase = 436.1 mm^3^ and 755.9 mm^3^, resp.). These findings suggest that it is difficult to reach a complete volume increase with bigger final volume expanders, probably due to higher pressure distribution to the underlying bone surface. However, this hypothesis needs to be confirmed in future studies, also comparing the volume increase results between different expander insertion approaches, that is, subperiosteal versus submucosal insertion techniques.

To summarize, STE might be a useful pre-augmentation approach specifically for vertical bone augmentation, as it results in high vertical bone gain with minimal post-surgical complications. The ideal clinical scenario for this specific application would be the need of vertical bone augmentation in the posterior mandible with limited amount of present soft tissues, consisting only of alveolar mucosa.

Findings of this report of four pilot cases must be interpreted with caution; volume analysis does not provide information on the actual volume changes in the tissues, as the post-expansion impressions were taken while expanders were still in situ. However, the volumetric analysis still gives an insight into the overall soft tissue volume changes; expansion pressure to the underlying bone surface, with subperiosteal large expanders, might prevent full soft tissue volume increase corresponding to the final expander volume.

The technique investigated in the present pilot cases still requires improvement for being considered predictable. Future clinical studies should also aim at comparing different expander insertion approaches as well.

## 6. Conclusions

Soft tissue expansion (STE) might be a beneficial pre-augmentation approach, especially in the vertical dimension. The ideal area for this specific application would be the posterior mandible with the presence of alveolar mucosa.

However, the presented technique still requires improvement before being applied routinely in everyday dental practice.

## Figures and Tables

**Table 1:** Volume gain analysis and bone fill calculations of the study participants.

Expansion zone	Initial expander volume	Final expander volume	Total expansion days	Expansion days as recommended by the manufacturer for the specific final volume	Soft tissue volume gain	Success of expansion	Augmentation dimension	Amount of bone fill in the vertical direction	Amount of bone fill in the horizontal direction
Right posterior mandible (Case 1)	0.25 ml (250 mm^3^)	1.3 ml (1300 mm^3^)	60 days	60 days	755.9 mm^3^	Successful	Vertical	8 mm	N/A
Anterior mandible (Case 2)	0.15 ml (150 mm^3^)	0.7 ml (700 mm^3^)	38 days	40 days	N/A	Late soft tissue expansion failure	Horizontal	N/A	Patient dropped out of the study after expansion failure
Right posterior mandible (Case 3)	0.05 ml (50 mm^3^)	0.35 ml (350 mm^3^)	10 days	40 days	N/A	Early soft tissue expansion failure	Horizontal	N/A	2 mm
Left posterior maxilla^∗^ (Case 4)	0.15 ml (150 mm^3^)	0.7 ml (700 mm^3^)	40 days	40 days	436.1 mm^3^	Successful	Horizontal	N/A	3 mm
Right posterior maxilla^∗^ (Case 4)	0.045 ml (45 mm^3^)	0.24 ml (240 mm^3^)	20 days	20 days	259.4 mm^3^	Successful	Horizontal	N/A	3 mm

N/A: not applicable. ^∗^Both sites were in the same patient (Case 4).

## Data Availability

All data is available in the manuscript.
